# Changes in the socio-demographic patterning of late adolescent health risk behaviours during the 1990s: analysis of two West of Scotland cohort studies

**DOI:** 10.1186/1471-2458-11-829

**Published:** 2011-10-26

**Authors:** Helen Sweeting, Caroline Jackson, Sally Haw

**Affiliations:** 1 MRC Social and Public Health Sciences Unit, 4 Lilybank Gardens, Glasgow, G12 8RZ, UK; 2 Scottish Collaboration for Public Health Policy and Research, MRC Human Genetics Unit Building, Western General Hospital, Crewe Road South, Edinburgh, EH4 2XU, UK; 3 Centre for Public Health & Population Health Research, School of Nursing, Midwifery and Health, University of Stirling, Stirling, FK9 4LA, UK

**Keywords:** Adolescent behaviour, time-trends, drinking behaviour, smoking, illicit drugs, sexual behaviour

## Abstract

**Background:**

Substance use and sexual risk behaviour affect young people's current and future health and wellbeing in many high-income countries. Our understanding of time-trends in adolescent health-risk behaviour is largely based on routinely collected survey data in school-aged adolescents (aged 15 years or less). Less is known about changes in these behaviours among older adolescents.

**Methods:**

We compared two cohorts from the same geographical area (West of Scotland), surveyed in 1990 and 2003, to: describe time-trends in measures of smoking, drinking, illicit drug use, early sexual initiation, number of opposite sex sexual partners and experience of pregnancy at age 18-19 years, both overall and stratified by gender and socioeconomic status (SES); and examine the effect of time-trends on the patterning of behaviours by gender and SES. Our analyses adjust for slight between-cohort age differences since age was positively associated with illicit drug use and pregnancy.

**Results:**

Rates of drinking, illicit drug use, early sexual initiation and experience of greater numbers of sexual partners all increased significantly between 1990 and 2003, especially among females, leading to attenuation and, for early sexual initiation, elimination, of gender differences. Most rates increased to a similar extent regardless of SES. However, rates of current smoking decreased only among those from higher SES groups. In addition, increases in 'cannabis-only' were greater among higher SES groups while use of illicit drugs other than cannabis increased more in lower SES groups.

**Conclusion:**

Marked increases in female substance use and sexual risk behaviours have implications for the long-term health and wellbeing of young women. More effective preventive measures are needed to reduce risk behaviour uptake throughout adolescence and into early adulthood. Public health strategies should reflect both the widespread prevalence of risk behaviour in young people as well as the particular vulnerability to certain risk behaviours among those from lower SES groups.

## Background

Many health-risk behaviours, including tobacco, alcohol and illicit drug use and 'risky' sexual behaviours develop during adolescence and are often maintained into adulthood, potentially affecting young people's current and future health and well-being in most high-income countries. This is particularly true in the UK, where the prevalence of these risk behaviours is higher than in similar countries [1].

Our understanding of time-trends in health-risk behaviours among young people largely stems from repeated cross-sectional school-based health and behaviour surveys [2-6]. Less is known about time-trends among *older *adolescents or young adults, among whom fewer data are routinely collected and where analyses may be limited by small numbers and the use of broad age categories such as 16-24 years [7-9], potentially masking differential trends in behaviours between those at either end of the age spectrum.

It is difficult to draw overarching conclusions about health-risk behaviour trends in an international context, because surveys have included different age groups and different behavioural measures. However, some patterns have emerged which are common to many middle- to high-income countries, particularly with respect to gender differences. Among younger adolescents, during the 1990s and into the early 2000s there was a convergence of males' and females' smoking behaviour [10,11], Indeed, in some northern and western European countries a female excess in smoking prevalence emerged [4,10]. Although in younger adolescence, males are more likely to drink alcohol and use cannabis, the gender gap in these behaviours also appears to have diminished in many countries [4,10]. These changing gender patterns may be partly due to more general changes in the lifestyles of younger adolescent males and females during this period [12]. There is greater heterogeneity in findings relating to sexual behaviour, with males more likely than females to have had sexual intercourse at age 15 in some countries, but not in others [10]. Although fewer data are available, results from a number of studies suggest that some of the changing gender patterns of health-risk behaviour observed among younger adolescents may also have occurred among older adolescents or young adults [13-15].

The evidence for social gradients in health-risk behaviours in adolescence is rather mixed. However, a comprehensive review conducted in 2007 found that among older adolescents, smoking was more likely among young people from lower socio-economic groups, but that there was little evidence of social gradients in alcohol or cannabis use [16]. The socio-economic patterning of sexual risk behaviour appears to vary between countries and according to the socio-economic measure used. However, there is evidence that lower socio-economic status (SES) and less family affluence are associated with early sexual initiation in some countries [17,18].

In the current study, we aimed to explore time-trends in the socio-demographic patterning of health-risk behaviours in older adolescents, using self-report data collected in the West of Scotland. When making comparisons of self-report data over time it is important to ask the same questions of socially and geographically comparable groups at every time point [19]. In this study, we compare data from older adolescents (18-19 years) from two studies that surveyed participants from exactly the same geographical area at two time points, 13 years apart, and in which a range of identical (or very similar) self-report health-risk behaviour measures were collected. We describe changes between 1990 and 2003 in smoking, drinking and illicit drug use and sexual risk behaviour, both overall and stratified according to gender and SES and examine the effect of changes over time on the patterning of these behaviours according to gender and SES. We have shown previously that the socio-demographic patterning of substance use differs according to the way in which particular substances are defined; for example, how detailed a definition of smoking (occasional, regular, heavier, etc.) is used [20], or when 'cannabis-only' users are distinguished from those who have used drugs other than cannabis [21]. The measures available within the current study allow us to examine whether changes over time are equivalent for two or more definitions of each behaviour. Additionally, given that age is associated with health-risk behavior uptake, we investigate the impact of the relatively small age differences between the cohorts on risk behaviour time-trends. Adjustment is also made, where appropriate, for gender and social class.

## Methods

### Study population

This study includes data from 18-19-year olds who participated in two studies in the West of Scotland: the *'Twenty-07 Study: Health in the Community' *(*'Twenty-07'*) [22] in 1990 and the *'11-16/16+ Study: Young People's Health' ('11-16/16+) *[23] in 2003. Both studies received approval from Glasgow University ethics committees.

*The Twenty-07 Study *began in 1987, when its youth cohort (one of three cohorts) was aged 15. The study was located in the Central Clydeside Conurbation, a predominantly urban area centred around Glasgow city. Cohorts were sampled via a two-stage clustered stratified random method based on postcode sectors (the primary sampling units) and individuals of appropriate ages within selected sectors [24]. The selection of individuals (in the case of the youth cohort, those born between 1^st ^March 1971 and 29^th ^February 1972) from primary sampling units was made possible by Strathclyde Region's Voluntary Population Survey, an annual census in which the age and sex composition of every household was ascertained. Targeted individuals were approached first by Strathclyde Regional Council to obtain consent to have their names passed to the study team and subsequently by the team to seek participation in the study. For the youth cohort, consent was required from both the young person and a parent. At baseline, a response rate of 65% of the issued sample was obtained, with no significant gender or social class differences compared with the population from which it was drawn [25]. At that stage, separate interviews were conducted with both the young people (aged 15) and their parents. In 1990, 908 (90%) of the original 1009 baseline participants (mean age 18 years 7 months, standard deviation [SD] ± 4 months; 430 males, 478 females) were interviewed in their homes by trained nurse interviewers using paper questionnaires. Loss to follow-up was slightly greater among respondents from lower SES backgrounds (reflected via parental economic activity, housing tenure and accommodation type), and an attrition-based weighting scheme was developed to compensate for this [26].

*The 11-16/16+ Study *cohort was recruited in 1994 during their final year of primary schooling. The sample, located like that of the *Twenty-07 Study*, in the Central Clydeside Conurbation, was designed to be representative at both primary and secondary school levels. Full details of the sampling strategy are available elsewhere [27], but briefly it involved a reverse sampling procedure comprising: (a) the random selection of 43 secondary schools, stratified by educational district, religious denomination (Catholic: non-denominational), and deprivation (proportion receiving clothing grant); (b) the random selection of 135 associated primary schools (similarly stratified) together with other primary schools making large numbers of placing requests to the selected secondary schools; and (c) the random selection of classes of 11 year-old pupils in each of these primary schools. The final eligible sample consisted of all pupils transferring to the 43 secondary schools, of whom 2586 (93%) participated in the baseline survey (age 11). At that stage, questionnaires were also completed by parents in respect of 2237 children. The cohort was followed up twice during secondary schooling and then post-school, in 2002-4 (henceforth 2003). At that stage, 1258 respondents (49% of the original cohort; 640 males, 618 females) were interviewed by trained interviewers using computer-assisted interviews. Interviews took place in survey centres set up in two locations at Glasgow University and at schools previously involved in the study, together with home visits for those unable or unwilling to travel elsewhere. Fieldwork for this stage took longer than anticipated, and was longer than that of *Twenty-07*, resulting in a sample which was slightly older and with a greater spread of ages (mean age 19 years 3 months, SD ± 6 months). Attrition-based weights were constructed, since at each study wave, loss to follow-up was greater among respondents from manual class backgrounds, those with lower teacher-rated ability and educational involvement, and those from reconstituted or lone-parent households [23]. Because these attrition-based weights were based on those present at all stages their effect is to reduce the size of the 2003 dataset to 1006 respondents.

Table 1 shows the gender and baseline social class characteristics of each study's baseline sample, alongside the characteristics of the 18-19 year-old non-responders and responders, as well as the group of weighted responders. In both studies, the effect of weighting was to reduce the proportion from a non-manual social class background. In the Twenty-07 Study (1990 dataset), differences between the baseline, age 18-19 responders and the age 18-19 weighted dataset were all small and non-significant. In the 11-16/16+ Study (2003) dataset, social class differences between the baseline (43.3% non-manual) and age 18-19 respondents (51.2% non-manual) were statistically significant (chi-square = 20.0, p < .001), however differences between the baseline and the age 18-19 weighted dataset (44.0% non-manual) were very small indeed (chi-square = 0.1, p = .702).

**Table 1:** Gender and social class within the Twenty-07 and 11-16/16+ studies-baseline samples, age 18-19 (non-)responders and age 18-19 weighted data.

	**Baseline sample**		**Age 18-19 non-responders**		**Age 18-19 responders**		**Age 18-19 responders-weighted data**	
	**N**	**%**	**N**	**%**	**N**	**%**	**N**	**%**
	
**Twenty-07 study (1990 dataset)**								
**Gender-age 15**								
Male	482	47.8	52	51.5	430	47.4	427	47.0
Female	527	52.2	49	48.5	478	52.6	481	53.0
*Total*	*1009*	*100.0*	*101*	*100.0*	*908*	*100.0*	*908*	*100.0*
**Social class-age 15**								
Non-manual	410	41.3	24	25.0	386	43.1	380	42.6
Manual	582	58.7	72	75.0	510	56.9	513	57.4
Missing	17	-	5	-	12	-	15	-
*Total*	*1009*	*100.0*	*101*	*100.0*	*908*	*100.0*	*908*	*100.0*
**11-16/16+ study (2003 dataset)**								
**Gender-age 11**								
Male	1335	51.6	701	52.4	634	50.8	505	50.2
Female	1251	48.4	636	47.6	615	49.2	501	49.8
*Total*	*2586*	*100.0*	*1337*	*100.0*	*1249*	*100.0*	*1006*	*100.0*
**Social class-age 11**								
Non-manual	1028	43.3	417	35.3	611	51.2	414	44.0
Manual	1348	56.7	765	64.7	583	48.8	527	56.0
Missing	210	-	155	-	55	-	65	-
*Total*	*2586*	*100.0*	*1337*	*100.0*	*1249*	*100.0*	1006 *	*100.0*

* Attrition-based weights based on those present at all four waves of the 11-16/16+ study reduce the size of the age 18-19 dataset to 1006 respondents

### Definitions

#### Behavioural measures

*Smoking*: in 1990, interviewers asked 'Do you smoke now, even if it is just occasionally?', and in 2003, 'Have you never smoked at all (not even a puff), have you ever tried (even if it was just a puff), are you an ex-smoker or do you smoke now?'. Responses to these questions were used to derive a dichotomous 'current smoker' variable. Smokers were also asked 'How many cigarettes do you smoke a day?' (in 1990) and 'On average, how many cigarettes do you smoke each week?' (in 2003), allowing the derivation of a heavier smoking variable (≥ 70 cigarettes per week).

*Drinking*: at both dates, the interviews included a past week drinking grid. In 1990 this asked about pints, glasses, bottles and measures of beer, lager, shandy, stout and cider; wine; fortified wine; spirits; and other drinks. The 2003 grid was more detailed reflecting the diversification of alcoholic beverages since 1990, and asked about pints, small, large and very large cans and bottles and small and large glasses of shandy; normal or strong beer, lager or stout; normal or strong cider; babycham; wine or champagne; cocktails, mixers, breezers or alcopops; spirits or liqueurs; (flavoured) schnapps; buckfast, eldorado or sanatogen; sherry, martini, taboo or port; MD20/20; and other drinks. From these, dichotomous variables were derived representing drinking over weekly recommended alcohol limits (hereafter referred to as 'excessive drinking': ≥ 22 units in the past week for males, ≥ 15 for females) [28] and 'binge drinking' (using the UK Office for National Statistics definition of ≥ 9 units on any day in the past week for males, ≥ 7 for females) [29].

*Illicit drug use*: respondents in both studies were provided with a list of illicit drugs comprising: cannabis; LSD; temazepam; tranquillisers; glues, sprays, gas, dry cleaning fluid; amphetamine; amyl or butile nitrite; heroin; methadone; temgesic; cocaine; crack; ecstasy; magic mushrooms; morphine or opium (the 1990 list also included other barbiturates and PCP). Respondents were asked if they had experience of any from the list, and, if applicable, whether they had taken it in the past year. Four variables were derived: ever use of any drugs, and past year use of: any drugs; cannabis-only (i.e. no other drugs-henceforth 'cannabis-only'); and any other drug(s) (including respondents taking other drugs *and *cannabis-henceforth 'other drugs').

*Early sexual initiation, number of sexual partners and pregnancy*: at both dates, respondents were asked 'How old were you when you first had sexual intercourse with someone of the opposite sex, or hasn't this happened?'. This was used to derive a dichotomous variable representing early sexual initiation (age ≤ 15 years vs ≥ 16 years or hasn't happened). Note that while all other data relate to *current *(1990 and 2003) behaviours, this was *recall *of sexual initiation by (approximately) 1987 for *Twenty-07 *and 1999 for *11-16/16+ *respondents. All who had hetero-sexual experience were asked how many opposite sex partners they had ever had sexual intercourse with and how many in the last year. They were also asked whether, at the time of the interview, they had ever made anyone, or been, pregnant.

#### Socioeconomic status

*Social class *was derived from head of household occupation. This information was collected at baseline in both studies, in *Twenty-07 *via parental interview, in *11-16/16+ *via parental self-completion questionnaire (supplemented, where necessary, by information provided by the pupils during interviews with research nurses which we have shown to be reliable [30]). Social class was dichotomised into: non-manual (NM, comprising: I-professional; II-managerial and technical; and IIINM-skilled non-manual occupations) and manual (M: IIIM-skilled manual; IV-semi-skilled; and V-unskilled).

*Carstairs-Morris deprivation categories *[31], were assigned to home postcodes obtained at age 15. The Carstairs-Morris index, developed for use in Scotland, is an area-based measure composed of four indicators representing material disadvantage (household overcrowding, male unemployment, social class IV or V, households with no car). The four indicators are combined to create a composite score which is then divided into seven separate categories ranging from very high to very low deprivation. For the purpose of analysis, scores were dichotomised into lower (categories 1-4) and higher (5-7) area deprivation.

### Analyses

We conducted analyses for each behavioural outcome using data weighted to compensate for attrition, restricting our analyses to those with social class data or, in analyses focusing on area deprivation, to those with deprivation category data (no respondent had missing gender or age data).

First, we examined associations between each risk behaviour (apart from early sexual initiation) and age at each date. (Since early sexual initiation was based on retrospective data, we would not expect it to be associated with age at interview.) We used logistic regression to obtain odds ratios (ORs) for each risk behavior according to a one month increase in age, with further logistic regression including interaction parameters to examine whether the association with age differed for 1990 vs 2003, males vs females; or respondents from non-manual vs manual backgrounds.

Analyses of changes in risk behaviour rates over time included:

• for the overall sample - cross-tabulations of risk behavior rates according to date, followed by logistic regression modeling to obtain unadjusted, age-adjusted, social class-adjusted and both age and social class-adjusted ORs for risk behaviour rates, comparing 2003 with 1990;

• for males and females separately - cross-tabulations of risk behavior rates according to date and logistic regression modeling to obtain age and social class-adjusted ORs for risk behaviour rates, comparing 2003 with 1990; followed by logistic regression modeling including age- and social class-adjusted interactions for gender by date, to examine whether the pattern of change between 1990 and 2003 differed for males compared with females;

• for respondents from non-manual and manual social class backgrounds separately - cross-tabulations of risk behavior rates according to date and logistic regression modeling to obtain age and gender-adjusted ORs for risk behaviour rates, comparing 2003 with 1990; followed by logistic regression modeling including age- and gender-adjusted interactions for social class by date, to examine whether the pattern of change between 1990 and 2003 differed for the two groups;

• as above, but for respondents from areas of lower and higher deprivation separately;

• finally, logistic regression modeling including the age-adjusted interactions of gender by social class by date, and gender by deprivation by date, to examine whether the time-trends differed for males and females from different SES backgrounds.

Because of the large number of analyses, all results described in the text as 'significant' are statistically significant at p < 0.01.

## Results

### Relationship between age and risk behaviours

As Table 2 shows, age was weakly but positively associated at both dates with smoking, experience of any drugs and past year experience of 'other drugs'. There was a much stronger relationship between age and both number of sexual partners and experience of pregnancy. A weak positive relationship between age and 'cannabis-only' in 1990 reversed to a negative relationship in 2003 (p-value of age by date interaction = 0.044). Only one of the interactions between age and each of date, gender or social class (a total of 33 analyses) reached more than this marginal level of significance, namely the age by gender interaction in respect of having had three or more sexual partners ever. Further analyses showed that the relationship between age and number of partners was stronger for females (OR for one month increase in age = 1.13, 95% CI = 1.10-1.16) than males (OR = 1.07, 95% CI = 1.04-1.09).

**Table 2:** Associations between reported rates of risk behaviours and age-ORs for one month increase in age when interviewed at each date (and significance of interactions between age and date, gender and social class).

	**1990**		**2003**		***(p-value of AGE by DATE*** ***Interaction)***	***(p-value of AGE by GENDER interaction)***	***(p-value of AGE by SOCIAL CLASS interaction)***
				
	**OR** **(95% CI)**	***p-value***	**OR** **(95% CI)**	***p-value***			
	
**Current smoker**	1.03 (0.99-1.07)	*0.089*	*1.02* *(0.99-1.04)*	*0.061*	*(0.582)*	*(0.286)*	*(0.954)*
Heavier smoking *	1.02 (0.98-1.06)	*0.378*	*1.01* *(0.98-1.04)*	*0.508*	*(0.710)*	*(0.044)*	*(0.220)*
**Excessive drinking ^†^**	0.99 (0.95-1.03)	*0.668*	*0.99* *(0.97-1.01)*	*0.491*	*(0.942)*	*(0.437)*	*(0.806)*
**Binge drinking ^‡^**	0.99 (0.95-1.02)	*0.479*	*0.99* *(0.97-1.01)*	*0.315*	*(0.896)*	*(0.132)*	*(0.976)*
**Use of any drugs**	1.03 (0.99-1.07)	*0.084*	*1.01* *(0.99-1.03)*	*0.359*	*(0.286)*	*(0.759)*	*(0.378)*
**Used any drug in the past year**	1.03 (0.99-1.07)	*0.115*	*0.99* *(0.97-1.01)*	*0.279*	*(0.058)*	*(0.902)*	*(0.503)*
**Used cannabis only in the past year (cannabis only) ^**	1.03 (0.98-1.08)	*0.257*	*0.97* *(0.95-0.99)*	*0.024*	*(0.044)*	*(0.261)*	*(0.716)*
**Used any other drug in the past year ('other drugs') ~**	1.03 (0.97-1.09)	*0.337*	*1.02* *(0.99-1.04)*	*0.225*	*(0.717)*	*(0.644)*	*(0.208)*
**Early sexual initiation ^#^**	N/A^¶^		N/A^¶^		*N/A*^¶^	*N/A*^¶^	*N/A*^¶^
**Three or more sexual partners ever**	1.08 (1.04-1.13)	*> 0.001*	*1.05* *(1.03-1.07)*	*> 0.001*	*(0.188)*	*(0 < .001)*	*(0.892)*
**Two or more sexual partners last year**	1.06 (1.02-1.11)	*0.007*	*1.02* *(0.99-1.04)*	*0.153*	*(0.064)*	*(0.564)*	*(0.273)*
**Ever (made) pregnant**	1.08 (1.00-1.16)	*0.046*	*1.06* *(1.03-1.09)*	*0.000*	*(0.689)*	*(0.800)*	*(0.794)*

* Defined as smoking 70+ cigarettes a week

^† ^Defined as ≥22 units in the past week for men and ≥15 units in the past week for women

^‡ ^Defined as ≥9 units on any day in the past week for men and ≥7 units on any day in the past week for women

^Defined as cannabis and no other drugs

~ Defined as any other drug(s) (with or without cannabis)

^# ^Defiined as sexual intercourse at age < 16 years

^¶ ^Data on early sexual initiation collected retrospectively so association with age when interviewed not applicable

OR = odds ratio, CI = confidence interval, NA = not applicable

### Changes in risk behaviour rates over time

Table 3 shows that overall, prevalence of all risk behaviours significantly increased between 1990 and 2003, apart from smoking, which remained stable, and heavier smoking, which significantly decreased. In 2003, almost half (46%) the respondents reported recent binge drinking, more than half (59%) reported some use of illicit drugs, more than a quarter (29%) reported first sexual intercourse under age 16 years, half (50%) had three or more sexual partners ever and one-third (35%) two or more in the last year. Adjustment for age resulted in slight attenuations in the ORs for 2003 compared with 1990 for smoking (thus somewhat magnifying the decrease in heavier smoking), use of any illicit drugs and past year use of 'other drugs'. Attenuation was much more marked in respect of number of sexual partners and experience of pregnancy, eliminating an apparent increase in rates of the latter between 1990 and 2003. In contrast, age-adjustment resulted in slight increases in the ORs for 2003 compared with 1990 for drinking and 'cannabis-only'. These results suggest the importance of accounting for the age difference (which was only 9 months) in the two cohorts. Adjustment for social class had no impact on the date difference in rates of any of these risk behaviours.

**Table 3:** Reported rates of risk behaviours at each date and age- and social class-adjusted ORs for 2003 compared with 1990.

	**1990**	**2003**	**1990**	**2003**	**Unadjusted**		**Adjusted for age**		**Adjusted for social class**		**Adjusted for age and social class**	
	
	**n/N**	**n/N**	**%** **(95%CI)**	**%** **(95%CI)**	**OR** **(95% CI)**	***p-value***	**OR** **(95% CI)**	***p-value***	**OR** **(95% CI)**	***p-value***	**OR** **(95% CI)**	***p-value***
	
**Current smoker**	300/892	319/939	33.6 (30.6-36.8)	34.0 (31.0-37.1)	1.02 (0.84-1.23)	*0.872*	0.85 0.67-1.08)	*0.181*	*1.02* *(0.84-1.24)*	*0.825*	*0.88* *(0.69-1.13)*	*0.323*
Heavier smoking *	200/891	145/940	22.5 (19.8-25.3)	15.4 (13.3-17.9)	0.63 (0.50-0.80)	*< 0.001*	0.57 (0.42-0.77)	*< 0.001*	*0.63* *(0.50-0.80)*	*< 0.001*	*0.60* *(0.44-0.81)*	*0.001*
**Excessive drinking ^†^**	183/892	301/926	20.5 (18.0-23.3)	32.5 (29.6-35.6)	1.87 (1.51-2.31)	*< 0.001*	1.99 (1.53-2.58)	*< 0.001*	*1.87* *(1.51-2.31)*	*< 0.001*	*1.96* *(1.51-2.55)*	*< 0.001*
**Binge drinking ^‡^**	308/891	427/927	34.6 (31.5-37.8)	46.1 (42.9-49.3)	1.62 (1.34-1.95)	*< 0.001*	1.76 (1.40-2.23)	*< 0.001*	*1.62* *(1.34-1.95)*	*< 0.001*	*1.77* *(1.40-2.24)*	*< 0.001*
**Use of any drugs**	291/892	553/941	32.6 (29.6-35.8)	58.8 (55.6-61.9)	2.94 (2.43-3.56)	*< 0.001*	2.62 (2.07-3.31)	*< 0.001*	*2.95* *(2.44-3.58)*	*< 0.001*	*2.66* *(2.10-3.37)*	*< 0.001*
**Used any drug in the past year**	216/891	375/940	24.2 (21.5-27.2)	39.9 (36.8-43.1)	2.07 (1.69-2.53)	*< 0.001*	2.11 (1.65-2.70)	*< 0.001*	*2.07* *(1.69-2.53)*	*< 0.001*	*2.09* *(1.63-2.68)*	*< 0.001*
**Used cannabis only in the past year (cannabis only) ^**	128/890	230/941	14.4 (12.2-16.8)	24.4 (21.8-27.3)	1.92 (1.51-2.44)	*< 0.001*	2.19 (1.64-2.93)	*< 0.001*	*1.92* *(1.51-2.43)*	*< 0.001*	*2.13* *(1.59-2.85)*	*< 0.001*
**Used any other drug in the past year ('other drugs') ~**	87/891	146/941	9.8 (8.0-11.9)	15.5 (13.4-18.0)	1.69 (1.28-2.25)	*< 0.001*	1.46 (1.03-2.06)	*0.033*	*1.70* *(1.28-2.26)*	*< 0.001*	*1.49* *(1.05-2.11)*	*0.024*
**Early sexual initiation ^#^**	139/889	267/938	15.6 (13.4-18.2)	28.5 (25.7-31.4)	2.15 (1.71-2.71)	*< 0.001*	*N/A*^¶^		*2.19* *(1.74-2.76)*	*< 0.001*	*N/A*^¶^	
**Three or more sexual partners ever**	212/887	464/925	23.9 (21.2-26.8)	50.2 (46.9-53.4)	3.21 (2.63-3.93)	*< 0.001*	2.15 (1.68-2.74)	*< 0.001*	*3.31* *(2.70-4.05)*	*< 0.001*	*2.28* *(1.78-2.92)*	*< 0.001*
**Two or more sexual partners last year**	158/890	331/934	17.8 (15.4-20.4)	35.4 (32.4-38.6)	2.54 (2.04-3.16)	*< 0.001*	2.11 (1.62-2.75)	*< 0.001*	*2.57* *(2.06-3.19)*	*< 0.001*	*2.18* *(1.67-2.85)*	*< 0.001*
**Ever (made) pregnant**	53/886	81/935	6.0 (4.6-7.7)	8.7 (7.0-10.6)	1.51 (1.05-2.16)	*0.026*	0.90 (0.57-1.41)	*0.643*	*1.53* *(1.07-2.20)*	*0.021*	*0.97* *(0.61-1.53)*	*0.885*

* Defined as smoking 70+ cigarettes a week

^† ^Defined as ≥22 units in the past week for men and ≥15 units in the past week for women

^‡ ^Defined as ≥9 units on any day in the past week for men and ≥7 units on any day in the past week for women

^Defined as cannabis and no other drugs

~ Defined as any other drug(s) (with or without cannabis)

^# ^Defiined as sexual intercourse at age < 16 years

^¶ ^Data on early sexual initiation collected retrospectively so adjustment for age when interviewed not applicable

OR = odds ratio for increase in behaviour rate between 1990 and 2003, CI = confidence interval, N/A = not applicable

### Changes over time by gender and SES

Analysis by gender showed increases in most risk behaviours were significantly more pronounced in females (Table 4). The exception was smoking; rates of current smoking remained stable for both genders, whilst the reduction in heavier smoking was largely confined to females. This resulted in the emergence of a male excess in heavier smoking in 2003 when rates among males and females were 19% and 11% respectively (p-value of age- and social class-adjusted gender difference < 0.001). Among females the age and social class-adjusted odds of excessive drinking in 2003 were almost four times higher (OR = 3.81, 95% CI = 2.47-5.87) than in 1990, while those of binge drinking were three times higher (OR = 3.06, 95% CI = 2.14-4.38). In contrast, in males, there were no significant increases in either excessive drinking (adjusted OR = 1.26, 95% CI = 0.89-1.77) or binge drinking (OR = 1.08, 95% CI = 0.78-1.50). Rates of all measures of illicit drug use (apart from males' past year use of 'other drugs') increased markedly for both genders, but more so among females. For example, the age- and social class-adjusted odds of past year use of any drugs in 2003 compared with 1990 were 1.73 (95% CI = 1.24-2.41) among males and 2.75 (95% CI = 1.85-4.08) among females. Compared with 1990, the social class-adjusted odds of early sexual initiation in 2003 were over four-fold higher (OR = 4.54, 95% CI = 3.03-6.80) among females, while increases among males were much smaller (OR = 1.38, 95% CI = 1.02-1.86). Gender differences in respect of increases in number of sexual partners were even more striking; thus the age and social class-adjusted odds of having had three or more sexual partners ever in 2003 compared with 1990 were 1.19 (95% CI = 0.85-1.67) among males, but 6.31 (95% CI = 4.12-9.67) among females. However, despite these increases in sexual risk behaviours, there was no significant increase in experience of pregnancy after adjustment for age and social class, among either males or females.

**Table 4:** Reported rates of risk behaviours at each date and age- and social class-adjusted ORs for 2003 compared with 1990, by gender.

	MALES			FEMALES			Age- and social class-adjusted odds ratio for 2003 compared with 1990				*(p-value of GENDER by DATE interaction-age and social class adjusted)*
	
	1990	2003		1990	2003		MALES		FEMALES		
	
Risk behaviour	% (95% CI)	% (95% CI)	*p-value*	% (95% CI)	% (95% CI)	*p-value*	OR (95% CI)	*p-value*	OR (95% CI)	*p-value*	
**Current smoker**	33.2 (28.9-37.8)	35.1 (30.9-39.5)	*0.549*	34.0 (29.9-38.4)	33.0 (28.9-37.5)	*0.747*	0.89 (0.62-1.25)	*0.492*	0.88 (0.62-1.24)	*0.464*	*(0.447)*
Heavier smoking *	22.3 (18.6-26.6)	19.4 (16.1-23.2)	*0.276*	22.6 (19.0-26.5)	11.3 (8.7-14.5)	*< 0.001*	0.79 (0.52-1.18)	*0.249*	0.43 (0.27-0.67)	*< 0.001*	*(0.005)*
**Excessive drinking^† ^**	31.8 (27.5-36.3)	38.3 (34.0-42.8)	*0.041*	10.4 (8.0-13.5)	26.5 (22.6-30.7)	*< 0.001*	1.26 (0.89-1.77)	*0.197*	3.81 (2.47-5.87)	*< 0.001*	*(< 0.001)*
**Binge drinking ^‡^**	49.6 (44.9-54.4)	51.8 (47.3-56.3)	*0.518*	21.1 (17.6-25.0)	40.3 (35.9-44.8)	*< 0.001*	1.08 (0.78-1.50)	*0.644*	3.06 (2.14-4.38)	*< 0.001*	*(< 0.001)*
**Use of any drugs**	43.9 (39.3-48.7)	66.3 (62.0-70.4)	*< 0.001*	22.6 (19.0-26.5)	50.9 (46.3-55.4)	*< 0.001*	2.15 (1.53-3.01)	*< 0.001*	3.40 (2.40-4.80)	*< 0.001*	*(0.084)*
**Used any drug in the past year**	35.3 (30.9-40.0)	49.7 (45.2-54.1)	*< 0.001*	14.4 (11.6-17.9)	29.8 (25.8-34.1)	*< 0.001*	1.73 (1.24-2.41)	*0.001*	2.75 (1.85-4.08)	*< 0.001*	*(0.123)*
**Used cannabis only in the past year ('cannabis-only') ^**	19.8 (16.2-23.8)	29.0 (25.1-33.2)	*0.001*	9.6 (7.2-12.6)	19.6 (16.2-23.4)	*< 0.001*	1.92 (1.31-2.81)	*0.001*	2.43 (1.53-3.85)	*< 0.001*	*(0.190)*
**Used any other drug in the past year ('other drugs') ~**	15.4 (12.3-19.2)	20.6 (17.3-24.5)	*0.044*	4.7 (3.1-7.0)	10.0 (7.6-13.1)	*0.002*	1.12 (0.73-1.72)	*0.616*	2.53 (1.35-4.73)	*0.004*	*(0.165)*
**Early sexual initiation ^#^**	24.8 (20.9-29.1)	30.3 (26.4-34.6)	*0.063*	7.5 (5.4-10.2)	26.5 (22.7-30.7)	*< 0.001*	1.38 ^¶^ (1.02-1.86)	*0.038 *^¶^	4.54 ^¶^ (3.03-6.80)	*< 0.001 *^¶^	*(< 0.001) *^¶^
**Three or more sexual partners ever**	41.2 (36.5-45.9)	54.6 (50.1-59.1)	*< 0.001*	8.5 (6.3-11.4)	45.7 (41.2-50.2)	*< 0.001*	1.19 (0.85-1.67)	*0.319*	6.31 (4.12-9.67)	*< 0.001*	*(< 0.001)*
**Two or more sexual** **partners last year**	29.9 (25.8-34.5)	44.1 (39.7-48.6)	*< 0.001*	6.8 (4.9-9.5)	26.4 (22.5-30.6)	*< 0.001*	1.34 (0.94-1.90)	*0.101*	5.24 (3.25-8.44)	*< 0.001*	*(< 0.001)*
**Ever (made) pregnant**	3.1 (1.8-5.2)	5.3 (3.6-7.7)	*0.110*	8.6 (6.4-11.5)	12.2 (9.5-15.5)	*0.071*	1.37 (0.58-3.20)	*0.472*	0.87 (0.50-1.51)	*0.623*	*(0.590)*

* Defined as smoking 70+ cigarettes a week

^† ^Defined as ≥22 units in the past week for men and ≥15 units in the past week for women

^‡ ^Defined as ≥9 units on any day in the past week for men and ≥7 units on any day in the past week for women

^Defined as cannabis and no other drugs

~ Defined as any other drug(s) (with or without cannabis)

^# ^Defiined as sexual intercourse at age < 16 years

^¶ ^Data on early sexual initiation collected retrospectively so age adjustment not applicable; adjusted for social class only.

OR = odds ratio for increase in behaviour rate between 1990 and 2003, CI = confidence interval

Although greater increases in all risk behaviours (other than smoking) among females led to reductions in gender differences by 2003, early sexual initiation was the only behaviour where the attenuated difference became statistically non-significant (p-value of age- and social class-adjusted gender difference: 1990 = < 0.001; 2003 = 0.111).

As Table 5 shows, most risk behaviour rates increased in both social class groups. The exception was smoking. Non-significant decreases in current smoking among respondents from non-manual backgrounds (age and gender-adjusted odds of smoking in 2003 compared with 1990 = 0.60, 95% CI = 0.41-0.89), but increases among those from manual backgrounds (OR = 1.13, 95% CI = 0.83-1.56), resulted in an emerging social class difference (p-value of age- and gender-adjusted class difference in 1990 = 0.089, in 2003 < 0.001). Adjustment for age and gender removed the apparent greater reduction in heavier smoking rates among manual class respondents. Adjustment also attenuated the observed increases in excessive and binge drinking among those from non-manual backgrounds (adjusted odds for 2003 compared with 1990 around 1.4 for both measures of drinking), while in the manual class group, the adjusted odds were around two for both excessive and binge drinking. These differential increases eliminated a marginal social class difference in excessive drinking seen in 1990. Age-adjusted odds for 2003 compared with 1990 for use of any illicit drugs and past year use of any drug were between two and three for both class groups. However, while the increase in past year 'cannabis-only' was somewhat greater among those from non-manual (adjusted OR = 2.48, 95% CI = 1.62-3.80) than non-manual (adjusted OR = 1.82, 95% CI = 1.21-2.73) backgrounds, the use of 'other drugs' increased only among those from manual backgrounds (adjusted OR = 1.97, 95% CI = 1.24-3.13), with no change in use in the non-manual group (adjusted OR = 0.98, 95% CI = 0.56-1.70). As a result, by 2003, a small excess in past year 'cannabis-only' had emerged among the non-manual class group (p-value of age- and gender-adjusted class difference = 0.039), and an excess of past year any drugs and 'other drugs' use emerged among those from manual backgrounds (p-values of age- and gender-adjusted class differences = 0.009 and 0.003, respectively). Finally, although the proportions reporting early sexual initiation, greater numbers of sexual partners and pregnancy were significantly higher among those from manual class backgrounds at both time-points, the magnitude of the increases in early sexual initiation and sexual partners between 1990 and 2003 was similar for both groups, while that for pregnancy was non-significant for both.

**Table 5:** Reported rates of risk behaviours at each date and age- and gender-adjusted ORs for 2003 compared with 1990, by social class.

	NON-MANUAL			MANUAL			Age- and gender-adjusted odds ratio for 2003 compared with 1990				*(p-value of SOCIAL CLASS by DATE interaction-age and gender adjusted)*
	
	1990	2003		1990	2003		NON-MANUAL		MANUAL		
	
Risk behaviour	% (95%CI)	% (95%CI)	*p-value*	% (95%CI)	% (95%CI)	*p-value*	OR (95% CI)	*p-value*	OR (95% CI)	*p-value*	
**Current smoker**	30.5 (26.0 - 35.4)	25.7 (21.7 - 30.2)	*0.137*	36.0 (31.9 - 40.4)	40.4 (36.3 - 44.7)	*0.150*	0.60 (0.41-0.89)	*0.011*	1.13 (0.83-1.56)	*0.437*	*(0.058)*
Heavier smoking *	16.6 (13.2 - 20.8)	11.8 (9.1 - 15.3)	*0.055*	26.7 (23.0 - 30.8)	18.2 (15.2 - 21.7)	*0.001*	0.52 (0.31-0.86)	*0.011*	0.63 (0.43-0.91)	*0.015*	*(0.737)*
**Excessive drinking ^†^**	23.7 (19.7 - 28.3)	32.4 (28.0 - 37.0)	*0.007*	18.0 (14.9 - 21.6)	32.6 (28.6 - 36.7)	*< 0.001*	1.48 (1.00-2.19)	*0.049*	2.52 (1.74-3.64)	*< 0.001*	*(0.047)*
**Binge drinking ^‡^**	36.0 (31.2 - 41.0)	44.4 (39.7 - 49.3)	*0.016*	33.5 (29.5 - 37.8)	47.4 (43.1 - 51.7)	*< 0.001*	1.40 (0.98-2.01)	*0.068*	2.11 (1.53-2.92)	*< 0.001*	*(0.108)*
**Use of any drugs**	32.4 (27.8 - 37.4)	54.6 (49.8 - 59.3)	*< 0.001*	32.8 (28.8 - 37.1)	62.1 (57.8 - 66.1)	*< 0.001*	2.36 (1.65-3.39)	*< 0.001*	3.03 (2.19-4.20)	*< 0.001*	*(0.102)*
**Used any drug in the past year**	26.2 (21.9 - 30.9)	40.4 (35.8 - 45.2)	*< 0.001*	23.0 (19.5 - 26.9)	39.5 (35.4 - 43.7)	*< 0.001*	2.04 (1.40-2.98)	*< 0.001*	2.18 (1.55-3.08)	*< 0.001*	*(0.373)*
**Used cannabis only in the past year ('cannabis-only') ^**	15.5 (12.2 - 19.6)	28.6 (24.4 - 33.1)	*< 0.001*	13.6 (10.9 - 16.9)	21.1 (17.8 - 24.7)	*0.002*	2.48 (1.62-3.80)	*< 0.001*	1.82 (1.21-2.73)	*0.004*	*(0.391)*
**Used any other drug in the past year ('other drugs') ~**	10.6 (7.9 - 14.2)	11.8 (9.1 - 15.3)	*0.594*	9.1 (6.9 - 12.0)	18.4 (15.3 - 21.9)	*< 0.001*	0.98 (0.56-1.70)	*0.933*	1.97 (1.24-3.13)	*0.004*	*(0.015)*
**Early sexual initiation #**	11.8 (8.8 - 15.5)	21.7 (17.9 - 25.9)	*< 0.001*	18.5 (15.3 - 22.1)	33.8 (29.9 - 37.9)	*< 0.001*	2.05 ^¶^ (1.39-3.03)	*< 0.001 ^¶^*	2.30 ^¶^ (1.71-3.08)	*< 0.001 *^¶^	*(0.630) ^¶^*
**Three or more sexual** **partners ever**	19.1 (15.4-23.3)	42.2 (37.6-47.1)	*< 0.001*	27.5 (23.8-31.5)	56.5 (52.2-60.8)	*< 0.001*	2.17 (1.47-3.20)	*< 0.001*	2.48 (1.77-3.49)	*< 0.001*	*(0.613)*
**Two or more sexual** **partners last year**	15.0 (11.8-18.9)	31.5 (27.2-36.1)	*< 0.001*	19.8 (16.6-23.5)	38.5 (34.4-42.7)	*< 0.001*	2.00 (1.31-3.05)	*0.001*	2.40 (1.66-3.46)	*< 0.001*	*(0.940)*
**Ever (made) pregnant**	3.0 (1.7 - 5.3)	5.6 (3.7 - 8.2)	*0.080*	8.2 (6.1 - 10.9)	11.1 (8.7 - 14.1)	*0.113*	1.77 (0.74-4.26)	*0.201*	0.79 (0.46-1.37)	*0.409*	*(0.266)*

* Defined as smoking 70+ cigarettes a week

^† ^Defined as ≥22 units in the past week for men and ≥15 units in the past week for women

^‡ ^Defined as ≥9 units on any day in the past week for men and ≥7 units on any day in the past week for women

^Defined as cannabis and no other drugs

~ Defined as any other drug(s) (with or without cannabis)

^# ^Defined as sexual intercourse at age < 16 years

^¶ ^Data on early sexual initiation collected retrospectively so age adjustment not applicable; adjusted for gender only.

OR = odds ratio for increase in behaviour rate between 1990 and 2003, CI = confidence interval

Analyses of respondents from areas of lower versus higher deprivation showed very similar results to those of social class in terms of patterns of changes in risk behaviour over time (Table 6). Differences between the deprivation groups were particularly marked in respect of past-year 'cannabis-only' (greater increases among the less deprived) and 'other drugs' (greater increases among the more deprived), resulting in emerging differences between those from more and less deprived areas by 2003 (p-value of age- and gender-adjusted deprivation area difference = 0.017 for 'cannabis-only' and < 0.001 for 'other drugs'). Patterns of change in early sexual initiation also differed by deprivation level. As a result, by 2003, a significant excess in early sexual initiation had emerged in those from areas of higher (33.9%) compared with lower (24.2%) deprivation (age- and gender-adjusted p-value = 0.002).

**Table 6:** Reported rates of risk behaviours at each date and age- and gender-adjusted ORs for 2003 compared with 1990, by deprivation category.

	LOWER DEPRIVATION			HIGHER DEPRIVATION			Age- and gender adjusted odds ratio for 2003 compared with 1990				*(p-value of DEPRIVATION by DATE interaction-age and gender adjusted)*
	
	1990	2003		1990	2003		LOWER DEPRIVATION		HIGHER DEPRIVATION		
	
Risk behaviour	% (95%CI)	% (95%CI)	*p-value*	% (95%CI)	% (95%CI)	*p-value*	OR (95% CI)	*p-value*	OR (95% CI)	*p-value*	
**Current smoker**	33.0 (29.1 - 37.1)	29.2 (25.4 - 33.4)	*0.201*	34.2 (29.4 - 39.3)	36.4 (32.1 - 41.0)	*0.514*	0.67 (0.48-0.95)	*0.022*	0.95 (0.67-1.35)	*0.773*	*(0.185)*
Heavier smoking *	19.9 (16.7 - 23.6)	12.9 (10.2 - 16.1)	*0.003*	26.3 (22.0 - 31.1)	17.3 (14.1 - 21.1)	*0.002*	0.49 (0.32-0.76)	*0.002*	0.54 (0.36-0.83)	*0.005*	*(0.963)*
**Excessive drinking^†^**	21.8 (18.5 - 25.6)	33.7 (29.7 - 38.0)	*< 0.001*	17.8 (14.2 - 22.1)	30.6 (26.4 - 35.1)	*< 0.001*	1.69 (1.18-2.40)	*0.004*	2.68 (1.78-4.04)	*< 0.001*	*(0.463)*
**Binge drinking ^‡^**	36.0 (32.0 - 40.2)	49.0 (44.6 - 53.4)	*< 0.001*	31.4 (26.7 - 36.4)	43.0 (38.4 - 47.7)	*0.001*	1.64 (1.19-2.26)	*0.003*	2.14 (1.49-3.08)	*< 0.001*	*(0.926)*
**Used of any drugs**	31.8 (28.0 - 35.9)	56.9 (52.5 - 61.1)	*< 0.001*	34.5 (29.7 - 39.6)	58.2 (53.6 - 62.7)	*< 0.001*	2.28 (1.65-3.15)	*< 0.001*	2.40 (1.69-3.41)	*< 0.001*	*(0.854)*
**Used any drug in the past year**	24.0 (20.5 - 27.8)	38.1 (33.9 - 42.4)	*< 0.001*	24.3 (20.1 - 29.0)	38.5 (34.1 - 43.1)	*< 0.001*	1.89 (1.34-2.67)	*< 0.001*	2.03 (1.40-2.96)	*< 0.001*	*(0.841)*
**Used cannabis only in the past year ('cannabis-only') ^**	13.8 (11.1 - 17.1)	27.2 (23.5 - 31.3)	*< 0.001*	15.0 (11.6 - 19.1)	19.1 (15.7 - 23.0)	*0.125*	2.63 (1.78-3.90)	*< 0.001*	1.53 (0.98-2.40)	*0.063*	*(0.040)*
**Used any other drug in the past year ('other drugs') **~	10.0 (7.7 - 12.9)	10.9 (8.4 - 13.9)	*0.636*	9.3 (6.7 - 12.8)	19.3 (15.9 - 23.3)	*< 0.001*	0.80 (0.48-1.33)	*0.380*	2.10 (1.27-3.47)	*0.004*	*(0.009)*
**Early sexual initiation #**	14.4 (11.7 - 17.7)	24.2 (20.7 - 28.2)	*< 0.001*	16.9 (13.4 - 21.2)	33.9 (29.7 - 38.5)	*< 0.001*	1.87 ^¶^ (1.36-2.57)	*< 0.001 *^¶^	2.63 ^¶^ (1.86-3.70)	*< 0.001 *^¶^	*(0.184) *^¶^
**Three or more sexual** **partners ever**	22.6 (19.2-26.3)	47.1 (42.8-51.6)	*< 0.001*	25.5 (21.3-30.2)	53.1 (48.4-57.8)	*< 0.001*	1.83 (1.30-2.57)	*< 0.001*	2.57 (1.76-3.71)	*< 0.001*	*(0.542)*
**Two or more sexual** **partners last year**	17.4 (14.5-20.9)	34.1 (30.0-38.4)	*< 0.001*	17.8 (14.2-22.0)	34.5 (30.2-39.0)	*< 0.001*	1.94 (1.35-2.80)	*< 0.001*	2.63 (1.75-3.96)	*< 0.001*	*(0.856)*
**Ever pregnant**	4.4 (3.0 - 6.6)	5.2 (3.6 - 7.6)	*0.542*	8.8 (6.3 - 12.3)	13.9 (10.9 - 17.4)	*0.028*	0.53 (0.25-1.15)	*0.109*	1.04 (0.60-1.81)	*0.894*	(*0.518)*

* Defined as smoking 70+ cigarettes a week

^† ^Defined as ≥22 units in the past week for men and ≥15 units in the past week for women

^‡ ^Defined as ≥9 units on any day in the past week for men and ≥7 units on any day in the past week for women

^Defined as cannabis and no other drugs

~ Defined as any other drug(s) (with or without cannabis)

^# ^Defined as sexual intercourse at age < 16 years

^¶ ^Data on early sexual initiation collected retrospectively so age adjustment not applicable; adjusted for gender only.

OR = odds ratio for increase in behaviour rate between 1990 and 2003, CI = confidence interval

Finally, for no behaviour was there evidence of significant interactions for either gender by social class by date, or gender by deprivation grouping by date, indicating that the pattern of change between males and females did not differ by SES.

## Discussion

We found a dramatic increase in a range of health-risk behaviours among older adolescents from the West of Scotland during the 1990s. Prevalence of illicit drug use increased among both males and females. Among females, alcohol use, early sexual initiation and experience of greater numbers of sexual partners also increased significantly, resulting in a gender convergence in patterns of risk behaviour. We are not aware of significant Scottish environmental and/or policy changes between 1990 and 2003. However, in 2006, Scotland introduced a ban on smoking in all public places, and Scottish survey data suggests that since 2003 there has been a reduction in current smoking among 16-24-year old males (rates of 32% in 2003 and 24% in 2009) but not females (29% at both dates) [8].

Our findings are consistent with results from previous studies which suggest that the gender gap in substance use has been diminishing among older adolescents or young adults as well as younger adolescents [13-15]. The increases in risk behaviours that we observed occurred over a remarkably short period of time but are consistent with the results of previous analyses of changes in substance use among the same cohorts at age 15 years. These also showed gender convergence in risk behaviour rates, largely reflecting changes in female lifestyles during this time [12]. Of particular concern is the marked increase in female risk behaviour, especially early sexual initiation and multiple sexual partners which are associated with greater risk of sexually transmitted disease and teenage pregnancy before 18 years [16]. The former, particularly chlamydia, is associated with infertility, the latter with adverse health, economic and social outcomes for mother and child [32]. A UK-wide survey of sexual attitudes and lifestyles similarly reported an increase in early sexual initiation among females up to the mid-1990s, after which the rate appeared to stabilise [18]. However, it is also striking that at the same time as the marked increases in these particular sexual risk behaviours, there was no significant change in experience of pregnancy. This suggests more (effective) use of contraception at the later date, consistent with UK studies which have found increases in consistent condom use in adults [33] and prescription of hormonal contraceptives to adolescent females [34].

This study also adds to evidence that the socio-demographic patterning of a particular substance may vary according to the measures used [20,21] and highlights the importance of examining whether time-trends are equivalent across all measures. In particular, it showed SES differences in patterns of increase for 'cannabis-only' compared with 'other drugs'. In a previous analysis of the *Twenty-07 Study *cohort (1990 dataset), we found that unlike 'other drugs', 'cannabis-only' use in late adolescence, was more likely among those from non-manual backgrounds in full-time education; a 'student effect' [21]. The increase in cannabis use over time among those from higher SES backgrounds is of concern. However, perhaps more so is that the prevalence of (arguably more risky) past year use of 'other drugs' doubled among those from lower SES backgrounds.

Alcohol use also increased between 1990 and 2003, but there was little difference in the prevalence of these measures between socioeconomic groups, suggesting a lack of socioeconomic gradient with respect to excessive alcohol consumption and binge drinking in young people of this age. This lack of association has been reported for younger adolescents, and, for some measures of alcohol use, older adolescents [16]. However, other measures of SES appear to have a different relationship with alcohol use, highlighting the complexity of these relationships. For example, higher income is associated with a greater frequency of drinking, and quantity of drinking is associated with education, with the less well-educated consuming more alcohol in a single session [16,35]. Interestingly, rates of current smoking were more differentiated by SES at the later date, following differential changes in smoking rates between 1990 and 2003 by SES. Although data on young adults are often not analysed separately from older adults, our findings are supported by English survey data which report a similar social gradient [36]. Our findings on sexual risk behaviour concur with reports from a UK national survey of sexual attitudes and lifestyles, which also found an association between parental SES and early sexual intercourse [18]. However, as with alcohol use, SES is not associated with all measures of sexual risk behaviour [17], and the association between other measures of SES, such as family affluence, and sexual risk behaviour varies by country [17,37].

One of the strengths of this study is its comparison of two cohorts of young people from exactly the same geographic area and life-stage, surveyed using (near) identical questions, 13 years apart. It examines changes over time in behaviour patterns in older adolescents, after school leaving, a life-stage for which few routine data are collected. The size and narrow age range of the cohorts allowed a more accurate determination of health-risk behaviours during later adolescence than is possible with existing surveys which collect data for much wider age bands. However, our analyses also highlight the impact of small increases in age on certain risky behaviours, even in older adolescence. This suggests the possibility of critical periods of susceptibility to the uptake of certain behaviours which may be masked when studies of young people employ wide age-bands. It also underlines the importance of controlling for even small age differences in comparisons such as ours, in order to avoid erroneous conclusions [38].

The study does, however, have some limitations. First, the follow-up rate in the *11-16/16+ Study *(2003) was quite low, with greater non-response and loss to follow-up among respondents from lower SES backgrounds. Although we accounted for this via weighted analyses, we may not have fully compensated for the differential loss to follow-up of adolescents with more 'risky' patterns of behaviour. However, this would have under- rather than over-estimated time-trends towards increased adoption of risky health behaviours. In a sensitivity analysis we compared the prevalence of risk behaviours in both cohorts using unweighted data, and found that in the *11-16/16+ Study *(2003), for some risk behaviours, the prevalence was slightly lower when using the unweighted data. This is unsurprising, given the differential loss to follow-up described above.

Second, although the questions included at each date were very similar, not all were identical. The 'heavier smoking' variable was based on a question about daily cigarette consumption in 1990, but weekly consumption in 2003, which might have reduced reporting accuracy, while the more detailed drinking grid in 2003 might have encouraged increased reporting. Parental occupational data, used to derive social class, were collected via parental interview in the earlier dataset, and via parental self-completion questionnaire supplemented, if necessary, by interview-based information provided by the respondents. However, we have shown this latter data to be reliable [30] and there is little reason to think that the different data-collection methods would impact in such a way as to produce bias.

Third, although data on risk behaviours were collected in a similar manner (one-to-one interviews) in both studies, thereby limiting the introduction of information bias through differential misclassification of behaviours between the two cohorts, interviewer-administered questionnaires such as ours have been shown to lead to under-reporting of behaviours compared with self-administered instruments [39]. Under-reporting through social desirability responses may have occurred. However, recall error (in terms of accurate recall of past behaviours) may have been less likely, especially regarding illicit drug use, since questions on this referred to both more recent ('past year') and 'ever' use [39]. Age at first sexual intercourse may be particularly prone to inconsistent reporting, although this has only been demonstrated in younger adolescents [40]. It is hard to find Scotland-wide data with which to compare health risk behaviour rates among the 1990 cohort. The first Scottish Health Survey of individuals aged 16-64, including questions on smoking and drinking, collected data in 1995 [41], while the first independent Scottish Crime Survey, including questions on drug use for 12-59 year olds (but only 232 respondents in the 16-19 year age group) was launched in 1993 [42]. The first British National Survey of Sexual Attitudes and Lifestyles of 16-59 year olds was conducted in 1990-91. This included 3377 16-24 year olds [43], but Scotland is not reported on separately, presumably since it comprised fewer than 10% of the overall sample. However, some comparisons *can *be made between health risk behaviour rates among the 2003 cohort and those found in other studies. When compared with self-completion data obtained from a Scottish sample of 15-year olds in 1998, rates of (recalled) early sexual initiation were lower in the *11-16/16+ *cohort among females (27% compared with 37%) but not males (31% compared with 33%) [3]. However, rates of alcohol and tobacco use were broadly in keeping with those of young adults reported in the 2003 Scottish Health Survey [44]. For example, 'excessive' alcohol consumption (≥ 22 units in the past week for males, ≥ 15 for females) was reported by 38% males and 26% females from our 2003 cohort and, by 31% males and 23% females aged 16-24 in the 2003 Scottish Health Survey; rates of current smoking were 35% (males) and 33% (females) among our cohort compared with 32% (males) and 29% (females) in the Scottish Health Survey. However, past-year illicit drug use rates (reported by 50% males and 30% females from our 2003 cohort) were considerably higher than those reported in the 2003 Scottish Crime Survey (27% males and 20% females aged 16-19) [45], perhaps reflecting higher rates of drug use in and around Glasgow than Scotland as a whole.

Finally, it is possible that there may have been greater under-reporting of some risk behaviours in the 1990 than the 2003 cohort, particularly those that were perhaps less "normative" in the late 1980s and early 1990s (such as early sexual initiation). The 1990 cohort were all interviewed at home, while the majority of interviews with the 2003 cohort were conducted in survey centres at Glasgow University or schools previously involved in the study. Since several of the health risk behaviours reported here might be regarded as sensitive, it is possible that a perceived lack of confidentiality or privacy in the home situation [39] might have resulted in under-reporting at the earlier date. However, this is unlikely to entirely explain the general increase in risk behaviour, particularly among females, observed in this study.

The data we report here reflect risk behaviours towards the beginning of the first decade of the 21^st ^century. However, a more recent (2008-2009) Scottish survey of 16-24 year-olds, albeit a wider age range, revealed no increase in levels of alcohol [8] or illicit drug [9] use, and, as noted earlier, decreases in smoking prevalence among males since the beginning of the 2000s [8]. A survey of Scottish 15 year-olds conducted in 2006 also reported no change in early sexual initiation rates since data were first collected in 1998 [3]. This suggests that the rapid increase in risky adolescent behaviour during the 1990s did not continue into the new century. Nevertheless, the rates we report are amongst the highest in Europe. Given the potential for adolescent risk behaviours to continue into adulthood and affect future health, our results are of significance for the current adult population.

## Conclusions

Comparison of two cohorts from the West of Scotland, surveyed in 1990 and 2003 showed that rates of drinking, illicit drug use, early sexual initiation and experience of greater numbers of sexual partners increased significantly over this time period. Greater increases among females resulted in attenuation, and for early sexual initiation, removal, of gender differences. Most rates increased to a similar extent regardless of SES. However, among those from higher SES groups, rates of current smoking decreased slightly and changes in illicit drug use appeared confined to 'cannabis only'. In contrast, among those from lower SES groups, current smoking rates did not decrease while use of illicit drugs other than cannabis increased.

The findings of this study have implications for researchers, policy makers and public health practitioners. The high prevalence of risk behaviour in mid- and late-adolescence indicates a need for improved, truly preventive, interventions, to more effectively reduce risk behaviour development in young people and subsequent adverse health, social and economic outcomes in adulthood. Public health policy and strategies should reflect both the widespread prevalence of risk behaviours in young people and the particular vulnerability to certain behaviours among those from more disadvantaged socio-economic groups, with combined investment in universal and targeted multi-faceted preventive approaches needed to equitably improve their health and wellbeing.

## Competing interests

The authors declare that they have no competing interests.

## Authors' contributions

All authors contributed to the analysis plan and questions addressed in the paper, and all read and approved the final manuscript. HS contributed to the design of *11-16/16+ *and its data collection, cleaned data from both studies and conducted the analyses, drafted and revised the paper. She is guarantor. CJ and SH drafted and revised the paper.

## Pre-publication history

The pre-publication history for this paper can be accessed here:

http://www.biomedcentral.com/1471-2458/11/829/prepub
